# Parenting Warmth and Strictness across Three Generations: Parenting Styles and Psychosocial Adjustment

**DOI:** 10.3390/ijerph17207487

**Published:** 2020-10-15

**Authors:** Oscar F. Garcia, Maria C. Fuentes, Enrique Gracia, Emilia Serra, Fernando Garcia

**Affiliations:** 1 Department of Developmental and Educational Psychology, Faculty of Psychology, University of Valencia, 46010 Valencia, Spain; oscar.f.garcia@uv.es (O.F.G.); emilia.serra@uv.es (E.S.); 2 Department of Methodology of the Behavioral Sciences, Faculty of Psychology, University of Valencia, 46010 Valencia, Spain; m.castillo.fuentes@uv.es; 3 Department of Social Psychology, Faculty of Psychology, University of Valencia, 46010 Valencia, Spain; enrique.gracia@uv.es

**Keywords:** parenting practices, warmth, strictness, parenting styles, generations, psychosocial adjustment

## Abstract

Recent emergent research is seriously questioning whether parental strictness contributes to children’s psychosocial adjustment in all cultural contexts. We examined cross-generational differences in parental practices characterized by warmth and practices characterized by strictness, as well as the relationship between parenting styles (authoritative, indulgent, authoritarian, and neglectful) and psychosocial adjustment in adulthood. Parenting practices characterized by warmth (affection, reasoning, indifference, and detachment) and strictness (revoking privileges, verbal scolding, and physical punishment) were examined. Psychosocial adjustment was captured with multidimensional self-concept and well-being (life satisfaction and happiness). Participants were 871 individuals who were members of three generations of Spanish families: College students (G3), their parents (G2), and their grandparents (G1). Results showed two different cross-generational patterns in parenting practices, with an increased tendency toward parental warmth (parents use more affection and reasoning but less indifference across generations) and a decreased tendency toward parental strictness (parents use revoking privileges, verbal scolding, and physical punishment less across generations). Interestingly, despite cross-generational differences in parenting practices, a common pattern between parenting styles and psychosocial adjustment was found: indulgent parenting was related to equal or even better self-concept and well-being than authoritative parenting, whereas parenting characterized by non-warmth (authoritarian and neglectful) was related to poor scores.

## 1. Introduction

Parental socialization is an adult-initiated process (parents or primary caretakers) by which the young person acquires the culture and the habits and values congruent with adaptation to that culture, so that young person become responsible members of their society. Parental socialization is over when the adolescent reaches the adult age [1,2]. In the study of parental socialization, scholars examine the influence of parents on children through two different parenting practices (theoretically independent or unrelated): those of warmth and those of strictness. The four parenting styles are defined by the combined effects of both warm and strict parenting practices: authoritative (warmth and strictness), indulgent (warmth without strictness), authoritarian (strictness without warmth) and neglectful (neither warmth nor strictness).

Traditionally, research conducted mainly in Anglo-Saxon contexts with European-American samples has consistently related authoritative parenting (i.e., warmth and strictness) to optimum psychosocial adjustment. However, there are serious doubts about the benefits of the combined effects of parental warmth and strictness (i.e., authoritative parenting) as the best parenting strategy for all cultural contexts. Additionally, although parenting and its impact on psychosocial adjustment is usually examined with adolescents, only some studies have examined the impact of parental socialization once adolescence is over, with adult children. Most of these studies have suggested cross-generational differences in parenting practices, but their findings emerge from non-normative families examining abusive parental practices (e.g., physical, emotional, and even sexual abuse). Within the same cultural context, it is generally argued that parenting practices change across historical periods and even that the impact of parenting on child psychosocial adjustment might be different depending on generation [3,4,5]. The present paper aims to examine in adult children of three generations of families (i) cross-generational differences in parenting practices, and (ii) which parenting style is related to the highest psychosocial adjustment.

### 1.1. Theoretical Framework

Variations in children’s and adolescents’ psychosocial adjustment are linked to differences in parental socialization [6,7,8,9,10]. Based mostly on the work of Baumrid (1971) [6] and Maccoby and Martin (1983) [11], in order to capture parental socialization, researchers have widely identified two independent (i.e., orthogonal) parental dimensions: warmth and strictness. Parental practices characterized by support and reasoning are grouped into a warmth dimension (also called responsiveness, acceptance, or involvement), whereas surveillance parenting practices are grouped into a strictness dimension (also called demandingness or supervision). The combination of these two orthogonal dimensions leads to four parenting styles: authoritative, characterized by warmth and strictness; indulgent, characterized by warmth but not strictness; authoritarian, characterized by strictness but not warmth; and neglectful, characterized by neither warmth nor strictness [7,11,12]. Parenting styles represent relational qualities between parents and their children, and they make it possible to capture the emotional family climate better than the isolated analysis of parental practices [7,13].

Since the early studies by Diana Baurind [6,14], research in Anglo-Saxon contexts with European-American samples (mostly white middle-class families) has traditionally identified authoritative parenting (i.e., warmth and strictness) as the best parenting strategy to foster psychosocial adjustment in the form of instrumental competence (i.e., behavior that is socially responsible and independent) and academic success. However, research conducted with other samples and in other cultural contexts does not support the idea that authoritative parenting is always associated with the best psychosocial adjustment of the children. In this regard, a growing body of research suggests that parenting’s influence on the children’s psychosocial adjustment can also vary as a function of the cultural context (for reviews, see García and Gracia, 2014 [8]; Garcia, Serra, Garcia, Martinez, and Cruise, 2019 [5]; Pinquart, and Gerke, 2019 [9]; Pinquart and Kauser, 2018 [15]).

For example, some studies from the United States with ethnic minority groups, such as African Americans [16,17], Chinese Americans [18,19], Hispanic Americans [20,21], or multiethnic Americans [22], as well as some studies with Arab families [23,24,25], found some benefits related to authoritarian parenting, suggesting that the authoritarian style is an appropriate parenting strategy. In addition, some studies that examined parenting styles in European and Latin American countries found that indulgent parenting (warmth but not strictness) was related to optimal child development. Indulgent parenting (also labelled as permissive, characterized by warmth but not strictness) was related to equal or even better psychosocial adjustment than authoritative parenting, whereas authoritarian parenting (strictness but not warmth) and neglectful parenting (neither warmth nor strictness) have consistently been related to the worst psychosocial adjustment [26,27,28].

In order to understand the differential impact of parental socialization on the psychosocial adjustment of children depending on the cultural background, researchers usually analyze the extent to which the same parental practices are used by families around the world [4,29,30,31,32]. Overall, frequent parenting practices are perceived as more culturally normative by children [30], so that parenting may have different consequences for children’s psychosocial adjustment depending on the extent to which parenting practices (e.g., love withdrawal or physical punishment) are normative within a culture [32,33,34,35,36]. It is usually argued that normative parental practices (i.e., those that are more frequent) tend to be perceived as fair and reasonable by children. For example, research findings reveal that corporal punishment has different effects on children across countries depending on the frequency of its use within a cultural context (for a discussion, see Gershoff et al., 2010 [32]).

### 1.2. Parenting across Generations

Although research has examined the extent to which different parental practices are used across families from different countries and cultural contexts, little research attention has been paid to analyzing whether, within the same cultural context, parental practices also vary in members of families from different generations. Within the same cultural context, it is generally argued that parenting practices change across historical periods [3,4,29,37,38]. Most research examining differences in the use of parenting practices across generations is based on studies analyzing generational differences in abusive parental practices, such as physical, emotional, and even sexual abuse in non-normative families (for a review, see Madigan et al., 2019 [39]). Fewer studies have examined parental practices across generations in normative families [38,40,41,42,43,44,45]. Overall, research findings regarding change in the parental dimensions of warmth and strictness across generations suggest a tendency toward an increase in parental warmth and a decrease in parental strictness [43,44], although it is not clear which specific practices of warmth and strictness are changing across generations [38,42]. For example, Zhou and colleagues (2018) [42] examined parenting practices of warmth (support and praise) and strictness (criticism, corporal punishment and control) across three generations of Chinese families (grandchildren, their mothers, and their grandmothers). The cross-generational parenting profile for the warmth dimension indicated an increased tendency toward parental practices of support (“Did your parents support your interests or talent?”) and praise (“Did your parents praise and encourage you a lot?”). In contrast, the cross-generational parenting profile for the strictness dimension revealed a decreased tendency toward parental practices of strictness, but only in the use of criticism or corporal punishment (“Did your parents criticize you? Did they ever use corporal punishment?”). However, in the strict parental practice of control (“Did your parents have many constraints?”), no cross-generational differences were found.

### 1.3. The Present Study

Although differences in the use of parenting practices across generations have been found, and these variations have been hypothesized to be associated with differences in children’s psychosocial functioning, this question has not been exhaustively tested empirically. The present study aims to examine cross-generational changes in parenting practices and the link between parenting styles and psychosocial adjustment in adulthood across three generations in a European country (i.e., Spain). Interestingly, an emerging body of research suggests that parental socialization could be related to long-term psychosocial adjustment beyond adolescence [1,46,47,48,49,50]. Findings from these studies revealed that differences in parental socialization showed a long-term, consistent, theoretically predictable pattern with psychological adjustment, not only in adolescent children, but also in adult children, including young, middle-aged, and older adults. Nevertheless, these studies offered evidence about the long-term impact of parental socialization, but without examining cross-generational differences in parenting practices that might play a crucial role in understanding the differential impact of parenting styles on psychosocial adjustment. Specifically, in the present study, we will examine: (i) cross-generational differences in parental practices characterized by warmth and practices characterized by strictness; and (ii) the relationship between parenting styles (i.e., authoritative, indulgent, authoritarian, and neglectful) and psychosocial adjustment in adulthood. Two sets of psychosocial adjustment outcomes will be analyzed: self-concept and wellbeing.

## 2. Materials and Methods

### 2.1. Sample and Procedure

It was estimated that a minimum of 768 participants were needed to conduct the study with a statistical power of 95% (1 − β = 0.95), setting the conventional limits on error rates in statistical inference (α = β = 0.05) and detecting medium-small effect sizes (*f* = 0.15) [51] between the four parenting styles and the psychosocial adjustment criteria [52,53,54,55]. The research was conducted at a large public university in southeastern Spain [56,57], data were collected from 871 individuals who were members of three-generation families: College students (G3), their parents (G2), and their grandparents (G1). A total of 184 middle-class families participated, and in each family, the participants were one college student (G3), both parents (G2) and at least one grandparent (G1). All participants (G1, G2, and G3) completed the questionnaires. During the course period, participants received information about the purpose of the study and signed an informed consent. Respondents were informed that participation was voluntary, that they were free to terminate their participation at any time, and that their responses to the questionnaires would be confidential. The mean age for the grandparent generation (G1) was 78.32 years (*SD* = 6.90; range 60 to 99; 182 females and 145 males). The mean age for the parent generation (G2) was 51.04 years (*SD* = 4.17; range 39 to 61; 184 females and 176 males), and the mean age for the college student generation (G3) was 22.73 years (*SD* = 1.76; range 20 to 29; 95 females and 89 males). All the college-age participants in this study (a) were Spanish, as were their parents and four grandparents; (b) they all participated voluntarily; (c) they were undergraduate students of psychology, pedagogy and teaching studies, and (d) they received some course credit for participating. With a study sample of 871 respondents, a sensitivity power analysis among the four parenting styles guaranteed the detection of a medium-small effect size of 0.140 (*f* = 0.140, α = 0.05, 1 − β = 0.95). All questionnaires were completed anonymously with Institutional Review Board approval.

### 2.2. Measures

#### 2.2.1. Parental Socialization

Parental socialization was captured with the Parental Socialization Scale (ESPA29) [58], a self-report instrument widely used to examine parenting practices and parenting styles. Parental warmth was captured with the ESPA-29 acceptance/involvement dimension, which included the practices of affection, reasoning, indifference, and detachment subscales (the latter two were negatively related to the dimension). Parental strictness was captured with the ESPA-29 strictness/imposition dimension, which included the subscales of revoking privileges, verbal scolding, and physical punishment. As in some previous studies, the following sentence was included in the instructions: “Here are some phrases or statements that describe how parents act with their children. Compare each statement to the way your mother and father treated you when you were a child” [1,49,59,60]. All the statements are in past tense. Previous studies used a similar procedure to evaluate parenting among adult children, once parental socialization is over [59,60].

The ESPA-29 scale is composed of 212 items (106 for each parent), all referring to 29 representative situations of daily family life, including 13 family norms compliance situations (e.g., “If I respected the established schedules in my house”); where respondents rate the frequency (from 1 = never to 4 = always) in which their parents used the parenting practices of affection (“He/she showed affection”) and indifference (“He/she seemed indifferent”); and 16 non-compliance situations (e.g., “If I broke or ruined anything in my house”), where respondents rate the frequency (from 1 = never to 4 = always) in which their parents used the parenting practices of reasoning (“He/she talked to me”), detachment (“It didn’t matter to him/her”), verbal scolding (“He/she scolded me”), physical punishment (“He/she hit me”), and revoking privileges (“He/she took something away from me”). The ratings on affection and reasoning subscales, together with the inverted ratings on indifference and detachment subscales, the ESPA-29 acceptance/involvement dimension, were averaged to obtain the parental warmth score; whereas parental strictness scores were obtained by averaging the ratings on verbal scolding, physical punishment and revoking privileges subscales, the ESPA-29 strictness/imposition dimension.

Taking into account the scores obtained on the warmth and strictness dimensions, families were classified according to the parenting style that characterizes them. For this purpose, the sample was dichotomized using the median split (i.e., Pc50), considering both dimensions simultaneously and also taking into account the sex and age of the participants [12,61,62]. Thus, authoritative families were those that scored above the median in both dimensions; indulgent families were those that scored below the median in the strictness/imposition dimension and above it in the acceptance/involvement dimension; authoritarian families were those that scored above the median on strictness/imposition and below it on acceptance/involvement; and, finally, neglectful families were those that scored below the median in both dimensions [55,63].

The adequate psychometric properties of the ESPA-29, as well as the orthogonality of the two main dimensions and its invariance for sex and age, have been demonstrated in studies across different countries, such as the United States [62], Portugal [64], Brazil [65], and Spain [66]. Cronbach’s alphas obtained in this study were 0.98 for acceptance/involvement and 0.98 for strictness/imposition. The Cronbach’s alphas for the subscales were the following: affection: 0.97, indifference: 0.96, reasoning: 0.97, detachment: 0.92, verbal scolding: 0.95, physical punishment: 0.97, and revoking privileges: 0.97.

#### 2.2.2. Psychosocial Adjustment

Self-concept was measured using the Multidimensional Self-Concept Scale AF5 [67]. It is based on the multidimensional and hierarchical theoretical model by Shavelson, Hubner, and Stanton (1976) [68]. It is composed of 30 items that assess five self-concept dimensions, with six items per dimension and a 99-point Likert scale (1 = strongly disagree to 99 = strongly agree): academic/professional (e.g., “My teachers (superiors) consider me an intelligent and hard-working person”), social (e.g., “I make friends easily”), emotional (e.g., reversed item, “A lot of things make me nervous”), family (e.g., “My family would help me in any kind of trouble”), and physical (e.g., “I like the way I look”). The AF5, originally validated with a sample of more than 6000 adolescents and adults, has good psychometric properties for both age groups [50,67,69,70]. Additionally, the AF5 scale is commonly used in studies with adults [1,50,71,72]. The penta-factorial structure of this instrument as well as its invariance for sex and age have been confirmed in several studies across different countries, such as the USA [73], Chile [74], Portugal [69], Brazil [75], Spain [76,77] and China [78]. Cronbach’s alphas obtained in this study were: academic, 0.89, social, 0.80, emotional, 0.83, family, 0.77, and physical, 0.73.

Well-being was captured through two indicators: life satisfaction and happiness. Life satisfaction, which is usually defined as the cognitive component of well-being, was captured with the Satisfaction with Life Scale, SWLS [79], made up of five items (e.g., “If I could live my life over, I would change almost nothing”) rated on a 7-point Likert scale (1 = strongly disagree to 7 = strongly agree). Cronbach’s alphas obtained in this study was 0.87. This instrument is one of the most widely used scales to measure life satisfaction [80,81,82,83]. Additionally, SWLS is commonly used in studies with adult participants [84]. Happiness, which is usually defined as the emotional component of well-being [85,86], was captured with a single item (“How happy are you with your life in general”), rated on an 11-point Likert scale (1 = completely unhappy to 11 = completely happy). This evaluation is commonly used with adult participants [87]. Previous studies reported the adequate psychometric properties of this approach [88,89,90].

#### 2.2.3. Data Analyses

To analyze cross-generational differences in warmth and strictness parental practices, a two-way multifactorial (3 × 2) multivariate analysis of variance (MANOVA) was applied, one to the four parenting variables characterized by warmth (i.e., affection, reasoning, detachment, and indifference) and the other to the three parenting variables characterized by strictness (i.e., revoking privileges, verbal scolding, and physical punishment). The two factors were the adult children’s generation (G1, G2, or G3) and sex (male or female). Follow-up univariate F-tests were conducted for all the sources of variation when multivariate statistically significant differences were found. Univariate significant results were followed by post hoc Bonferroni comparisons of all the possible pairs of means.

To analyze the relationship between parenting styles and psychosocial adjustment in adulthood, a three-way multifactorial (4 × 3 × 2) multivariate analysis of variance (MANOVA) was applied to two sets of psychosocial adjustment outcome variables (self-concept and well-being). The three factors were parenting styles (authoritative, authoritarian, indulgent, and neglectful), adult children’s generation (G1, G2 or G3), and sex (male or female). The significant sets of psychosocial adjustment outcome variables in the MANOVA were then analyzed by using univariate *F*-tests and, significant differences in the univariate *F*-tests were after examined using the post-hoc Bonferroni test [91,92,93].

## 3. Results

### 3.1. Parenting Style Groups

Adult children from the three generations (i.e., G1, G2, and G3) were classified into one of the parenting style families (i.e., indulgent, authoritative, authoritarian, or neglectful; Table 1). The authoritative parenting group contained 219 participants (25.1%), with high warmth, *M* = 3.37 and *SD* = 0.35, and high strictness, *M* = 3.37 and *SD* = 0.30; the indulgent group had 217 (24.9%), with high warmth, *M* = 3.44 and *SD* = 0.32, but low strictness, *M* = 1.56 and *SD* = 0.25; the authoritarian group contained 219 (25.1%), with low warmth, *M* = 2.56 and *SD* = 0.30, and strictness, *M* = 2.28 and *SD* = 0.35; and the neglectful family contained 216 (24.8%), with low warmth, *M* = 2.54 and *SD* = 0.40, and low strictness, *M* = 1.50 and *SD* = 0.22.

### 3.2. Cross-Generational Differences in Parental Practices

Previously, since the data are clustered, with the three generations having been sampled within families, we applied preliminary nested multifactorial MANOVAs [94,95], with the 184 families as random factors for each set of outcomes as dependent variables (i.e., warmth and strictness practices). The MANOVA for the set of warmth parental practices did not yield statistically significant effects of family, Λ = 0.027, *F*(2188.0, 1274.9) = 0.86, *p* > 0.05, neither did the MANOVA for the set of strictness parental practices, Λ = 0.056, *F*(1641.0, 958.0) = 0.94, *p* > 0.05.

The results for the MANOVA conducted with the four parental warmth practices (i.e., affection, reasoning, indifference, and detachment) yielded a significant main effect for the generation, Λ = 0.937, *F*(8, 1730.0) = 7.15, *p* < 0.001. Moreover, the results for the MANOVA conducted with the three parental practices characterized by strictness (i.e., revoking privileges, verbal scolding, and physical punishment) showed a significant main effect for the generation, Λ = 0.857, *F*(6, 1732.0) = 23.11, *p* < 0.001.

**Figure 1:**
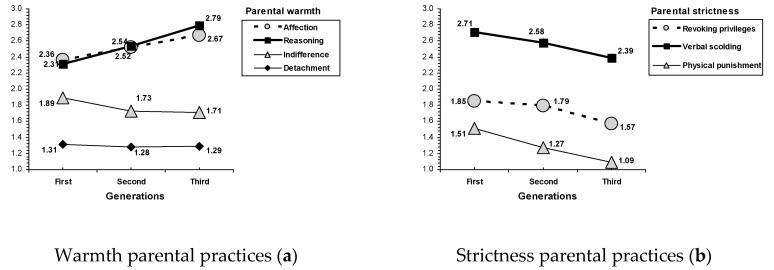
Parenting across generations for parental practices of (**a**) warmth, and (**b**) strictness.

Parental practices characterized by warmth have increased in families across generations (see Table 2, and Figure 1). Parents in the first generation tended to use less affection than in the second generation (G2) and the third generation (G3). Interestingly, according to the results of the *F* statistic, the biggest differences in parental warmth practices across the three generations were found in reasoning, *F*(2, 2868) = 23.53, *p* < 0.001, with an increasing tendency. In particular, parents in the second generation G2) use more reasoning than those in the first generation (G1), whereas parents in the third generation (G3) use higher reasoning than those in the second generation (G2). Similarly, the use of indifference (a parental practice characterized by low parental warmth) tends to decrease with the generations, with less detachment in the second generation (G2) and the third generation (G3) than in the first generation (G1).

By contrast, in the case of parental practices characterized by strictness, a decreasing tendency was found across generations (see Table 2, and Figure 1). The use of revoking privileges by parents is lower in the third generation (G3) than in the second generation (G2) and the first generation (G1). A similar decreasing tendency appeared for verbal scolding; parents in the second generation use less verbal scolding than in the first generation (G1), although parents in the third generation (G3) use less verbal scolding than in the second generation (G2). Interestingly, the biggest differences across the three generations was found in parental practices characterized by strictness, *F*(2, 865) = 65.77, *p* < 0.001. The use of physical punishment tends to decrease across generations. The highest use of physical punishment corresponded to parents from the first generation (G1), the lowest corresponded to those from the third generation, and parents from the second generation (G2) were in the middle position.

### 3.3. Parenting Styles and Psychosocial Adjustment

Previously, since the data are clustered, with the three generations having been sampled within families, we applied preliminary nested multifactorial MANOVAs [94,95], with the 184 families as random factors and the set of outcomes of psychosocial adjustment as dependent variables (i.e., self-concept and well-being). The MANOVA for the set of psychosocial adjustment did not yield statistically significant effects of family, Λ = 0.001, *F*(3829.0, 2219.7) = 1.04, *p* > 0.05.

Results of the MANOVAs yielded statistically significant main effects for parenting style, Λ = 0.795, *F*(21, 2415.4) = 9.58, *p* < 0.001, sex, Λ = 0.953, *F*(7, 841) = 5.93, *p* < 0.001, and generation, Λ = 0.854, *F*(14, 1682) = 9.87, *p* < 0.001. In addition, significant interaction effects were obtained between parenting style and generation, Λ = 0.886, *F*(42, 3948.1) = 2.45, *p* < 0.01, and sex and generation, Λ = 0.970, *F*(14, 1682) = 1.84, *p* < 0.05, (see Table 3).

Overall, the results for main effects of the parenting styles showed that indulgent parenting was related to equal or even better psychosocial adjustment than the authoritative style (see Table 4), whereas the worst psychosocial adjustment corresponded to parenting characterized by a lack of warmth (i.e., authoritarian and neglectful styles). Adult children from indulgent families scored higher than those from authoritarian and neglectful families on academic/professional self-concept. Additionally, adult children with authoritative parents obtained higher scores than those with authoritarian parents. On physical self-concept, adult children from authoritative, indulgent, and neglectful families scored significantly better than their peers with authoritarian parents. Finally, adult children with authoritative and indulgent parents obtained higher scores on happiness than those from authoritarian families (see Table 4).

**Figure 2:**
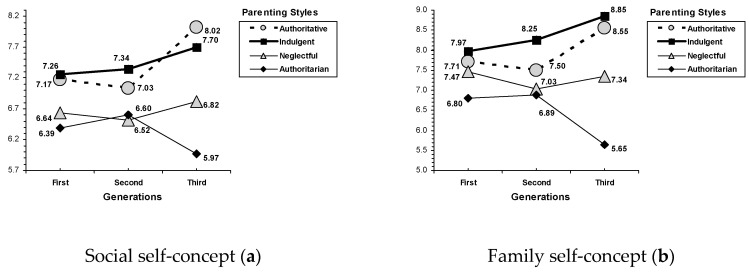

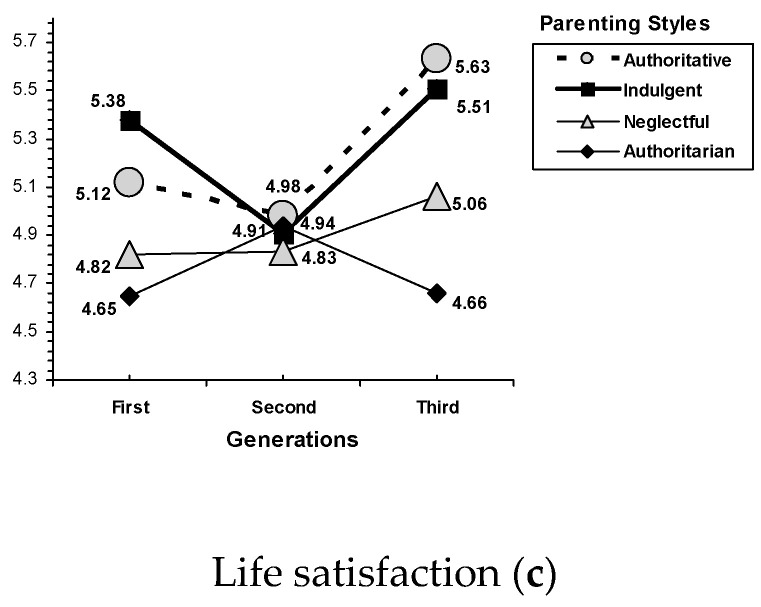
Interactions of parenting style by generation. (**a**) Social self-concept, (**b**) family self-concept, (**c**) life satisfaction.

Moreover, an interaction effect between parenting styles and the children’s generation was found on social self-concept, *F*(6, 847) = 2.68, *p* = 0.014, family self-concept, *F*(6, 847) = 7.72, *p* < 0.001, and life satisfaction, *F*(6, 847) = 2.89, *p* = 0.009. Once again, indulgent parenting and authoritative parenting were significantly related to better results than the authoritarian and neglectful parenting styles across children’s generations (see Figure 2). Whereas neglectful parenting showed mixed results, especially in adult children from the first generation (e.g., on family self-concept); authoritarian parenting was consistently related to the worst results across the generations, with adult children from the third generation showing the lowest scores on all the outcomes. Specifically, on social self-concept, adult children from indulgent and authoritative families scored significantly better than those from authoritarian and neglectful families in the first and third generations. Regarding the second generation, adult children with indulgent parents scored significantly better than those from authoritarian and neglectful families (see Figure 2, section A). On family self-concept, adult children with indulgent, authoritative, and neglectful parents obtained higher scores than those with authoritarian parents in the first generation. In the second generation, adult children from indulgent families scored significantly better than those from authoritative, authoritarian, and neglectful parents; whereas adult children with indulgent and authoritative parents obtained higher scores than those with authoritarian parents in the third generation (see Figure 2, section B). Finally, on life satisfaction, adult children from indulgent families scored significantly better than those from authoritarian and neglectful families in the first generation; and adult children with indulgent and authoritative parents obtained higher scores than those with authoritarian and neglectful parents (see Figure 2, section C).

**Figure 3:**
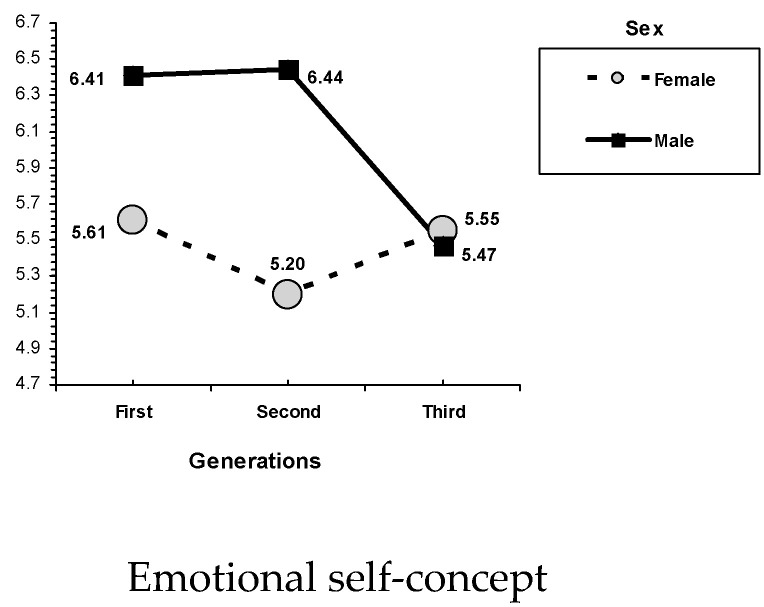
Interactions of sex and generation. Emotional self-concept.

Results revealed some sex and age related differences in self-concept and psychological adjustment (see Table 5). Regarding sex-related differences, results showed that males scored significantly higher than females on emotional and physical self-concept. In the case of generation-related differences, children from the first and second generations obtained higher scores on academic/professional self-concept than those from the third generation. On physical self-concept, adult children from the second and third generations scored better than adult children from the first generation. On life satisfaction, adult children from the first generation scored better than those from the third generation. Finally, adult children from the third generation showed greater happiness than those from the first and second generations. In addition, an interaction effect between sex and children’s generation was found on emotional self-concept, *F*(2,847) = 5.66, *p* = 0.004. Females from the first generation reported higher emotional self-concept than the males from the same generation. In the third generation, no sex-related differences were found (see Figure 3). Sex-related differences indicated that females showed higher family self-concept than males.

## 4. Discussion

The present study examines cross-generational differences in parental practices characterized by warmth (warmth, reasoning, indifference, and detachment) and strictness (revoking privileges, verbal scolding, and physical punishment), and the links between parenting styles (i.e., authoritative, indulgent, authoritarian, and neglectful) and psychosocial adjustment in adulthood in a community sample of Spanish adult children from three family generations. Overall, results showed cross-generational differences in parental practices characterized by warmth and strictness across generations. Importantly, despite these variations in the extent to which parental practices are used, the relationship between parenting styles and psychosocial adjustment (self-concept and well-being) showed a common pattern. The indulgent style (warmth but not strictness) was related to equal or even better results on psychosocial adjustment outcomes than authoritative parenting (warmth and strictness), whereas parenting characterized by lack of warmth (authoritarian and neglectful) was associated with poor psychosocial adjustment.

An important contribution of the present study is that the main findings offer a clear pattern of cross-generational variations in parental practices. Specifically, parents tend to be warmer and more involved with their children and less strict and imposing over the generations. A cross-generational pattern of increasing parental warmth was found. Parents in the second generation and the third generation use affection more and indifference less than in the first generation, and parents show the greatest use of reasoning in the first generation, the lowest in the third generation, and an intermediate amount in the second generation. By contrast, a cross-generational pattern of decreasing parental strictness was found. Parents in the first generation tend to use the revoking privileges strategy less than in the second generation and the third generation, and parents also show the greatest use of verbal scolding and physical punishment in the first generation, the lowest in the third generation, and an intermediate amount in the second generation. The findings of this study confirmed the cross-generational effects of parenting found by Olsen and colleagues (1999) [43] with a single global measure for warmth and strictness, but extending the evidence to seven specific parental practices rather than a single measure for each main dimension.

Another crucial finding of the present study is that, beyond the cross-generational differences in parental practices, those adult children who were raised by indulgent parents (warmth but not strictness) reported equal or even higher psychosocial adjustment than their peers from authoritative households, whereas those raised in neglectful and authoritarian homes were consistently associated with the poorest psychosocial adjustment. Overall, on multidimensional self-concept, parenting characterized by warmth (i.e., indulgent and authoritative) is related to better results than non-warm parenting (i.e., authoritarian and neglectful), although only indulgent parenting is consistently related to the highest self-concept. Furthermore, adult children from the second generation who characterized their parents as indulgent reported more family and social self-concept than their peers from authoritative families. Similarly, warm parenting styles (i.e., indulgent and authoritative) were positively related to multidimensional well-being in the life satisfaction and happiness domains, whereas non-warm parenting (i.e., indulgent and authoritative) offered poor benefits in terms of well-being.

On the other hand, findings from the present study indicate that the indulgent parenting style (i.e., warmth but not strictness) is an optimal strategy to foster psychosocial adjustment in children in the European cultural context, thus confirming evidence from previous studies conducted in European and South American countries, while extending it to three family generations of Spanish children and other developmental outcomes such as life-satisfaction. Children with indulgent parents obtained equal or even better adjustment and competence than those from authoritative families (warmth but not strictness) on several criteria such as self-concept [26,28], psychosocial maturity [1], internalization of values [50], protection against bullying and cyberbullying [96], psychological adjustment [49], child-to-parent violence [97], social and environmental values [98,99], protection against alcohol [95,100] and other drugs [101,102,103], school and sport achievement and academic adjustment [104,105,106].

Nevertheless, findings from the present study do not agree with some evidence from other cultural contexts where parental strictness is a necessary component of parental socialization in order to obtain children with good psychosocial adjustment. In middle-class European-American families, strictness is a necessary component of parental socialization, combined with parental warmth (i.e., authoritative parenting). For example, children with indulgent parents have greater academic self-conceptions and report less somatic distress (in the same way as their peers from authoritative homes), but they fail in their school orientation and indicate school misconduct (in contrast with children from authoritative households) [55,63]. In the same way, strict parenting is also necessary, even without parental warmth (i.e., authoritarian parenting), in families of ethnic minorities in the United States [16,18] or Arabic countries [23,25]. According to the present results examining three generations of Spanish families, the parental strictness component not only seems to be unnecessary, but it could also be negative for psychosocial adjustment because children from indulgent homes have equal or even greater self-conceptions and well-being than those from authoritative families.

Another crucial finding from the present study is that our results do not agree with the idea that cross-generational differences in the use of parental practices from one generation to another change the way these practices influence the children’s psychosocial adjustment (e.g., if physical punishment is more common or normative during a generation, it will not have negative influences, whereas if this parenting practice is no longer used, it will be likely to have negative impact on another generation). For example, parents in the first generation use the greatest strictness and imposition (including practices such as physical punishment and revoking privileges) and the poorest warmth and involvement (including less use of affection and reasoning, and greater use of indifference). However, despite the greater strictness and lower warmth in children from the first generation, and to a lesser extent in the second generation compared to the third generation, even children from the first generation raised in authoritarian families showed poorer psychosocial functioning. The authoritarian parenting style is consistently related to the lowest self-concept and well-being, regardless of the generation.

The long-term impact of parental socialization on the psychosocial adjustment of adult children confirms some previous research, but also extends the evidence to family generations using the same theoretical framework with four parenting styles [1,46,47,48,49,50]. For instance, prospective evidence from the MRC National Survey of Health and Development revealed a long-term impact of the childhood environment on the mental wellbeing at 60–64 years old [47], and childhood adversities and the home atmosphere were associated with adjustment in old age based on the Helsinki birth cohort study [107]. In the same line, parental warmth was associated with coping and well-being in adulthood using three waves of longitudinal data across 20 years from the national survey of Midlife Development in the United States [108]. A prospective longitudinal design revealed that the effects of parenting practices, both positive and negative, persisted well into mid-adulthood [109], and psychological outcomes in middle adulthood were linked to the mother’s child-rearing attitudes in early childhood, based on longitudinal evidence from the British Cohort Study (BCS70) [110].

In addition, results of this study agree with some previous studies about differences between demographic variables and psychosocial adjustment. Overall, older adults reported lower academic/professional and physical self-concept than young and middle-aged adults [1] but older adults also tended to have greater well-being (e.g., life-satisfaction and happiness) than young adults [111,112]. Females usually report greater family self-concept, but less emotional self-concept, than males [73,75].

The present findings should be interpreted with caution. We cannot exclude either causal relations between variables or third-variable explanations, although the sample is relatively similar in demographic traits, making third-variable accounts less likely. Conclusions about the present findings should be considered preliminary due to the absence of longitudinal or experimental data. Parents’ behavior was obtained through children’s reports instead of parents’ reports, although similar results were found with different methods of data collection. The present study offers interesting evidence about the variations in parental practices characterized by warmth and strictness across family generations, also considering the relationship between parenting styles and psychosocial adjustment. The present study examines parenting in middle-class families, so future studies should test the impact of parenting in other socioeconomic settings. Additionally, future studies should continue to examine cross-generational differences in parental socializations and their impact on children’s adjustment

## 5. Conclusions

The present work also addressed main gaps in previous findings examining the linkage between parenting across generations and its impact on psychosocial adjustment. Most of the previous studies have focused on non-normative families, examining strict parental practices such as physical, emotional, and even sexual abuse [39]. However, less is known about the cross-generational differences in strict parental practices such as physical punishment, which, applied in the general emotional context of normative families (i.e., authoritarian parenting style), is quite different from other forms of harsh discipline such as physical abuse [113,114] (for a review, see Baumrind, Larzelere, and Cowan, 2002 [115]). The present findings, using a contextual [7] and situational [116] approach to capture parenting, revealed different patterns across generations in the parental practices of warmth (with an increasing tendency) and strictness (with a decreasing tendency). In contrast with some previous research, our study provides evidence by using a parenting styles framework that captures overall long-term parenting characteristics that integrate and organize the specific parenting practices of warmth and strictness. Furthermore, the impact of parenting was examined using the same psychosocial adjustment outcomes (self-concept and well-being) and seven indicators. The results confirm previous evidence about the link between parenting styles and long-term psychosocial adjustment outcomes, extending evidence to children raised by Spanish parents from three different family generations.

## Figures and Tables

**Table 1:** Numbers of Cases in Parenting Style Groups, Mean Scores, and Standard Deviations on Main Measures of Parental Dimensions.

	Total	Authoritative	Indulgent	Authoritarian	Neglectful
Frequency	871	219	217	219	216
Percent	100.0	25.1	24.9	25.1	24.8
Warmth					
*Mean*	2.98	3.37	3.44	2.56	2.54
*SD*	0.54	0.30	0.32	0.30	0.40
Strictness					
*Mean*	1.89	2.23	1.56	2.28	1.50
*SD*	0.47	0.35	0.25	0.35	0.22

**Table 2:** Means and (standard deviations) in parental practices of warmth and strictness across generations.

	G1	G2	G3	*F*(2, 2868)
Parental Warmth				
Affection	2.36 ^2^ (0.83)	2.52 ^1^ (0.76)	2.67 ^1^ (0.78)	9.36 ***
Reasoning	2.31 ^3^ (0.83)	2.54 ^2^ (0.73)	2.79 ^1^ (0.72)	23.53 ***
Indifference	1.89 ^1^ (0.73)	1.73 ^2^ (0.62)	1.71 ^2^ (0.65)	6.11 **
Detachment	1.31 (0.38)	1.28 (0.30)	1.29 (0.35)	0.54
Parental Strictness				
Revoking Privileges	1.85 ^1^ (0.67)	1.79 ^1^ (0.58)	1.57 ^2^ (0.53)	13.02 ***
Verbal Scolding	2.71 ^1^ (0.66)	2.58 ^2^ (0.63)	2.39 ^3^ (0.61)	15.50 ***
Physical Punishment	1.51 ^1^ (0.55)	1.27 ^2^ (0.37)	1.09 ^3^ (0.20)	65.20 ***

Note: Bonferroni Test α = 0.05; ^1^ > ^2^ > ^3^, ** *p* < 0.01, *** *p* < 0.001.

**Table 3:** Multivariate analysis of variance (MANOVA) factorial (4 ^a^ × 2 ^b^ × 3 ^c^) for psychosocial adjustment (self-concept and well-being).

Source of Variation	Λ	*F*	*Df*_between_	*Df*_error_	*P*
(A) Parenting Style ^a^	0.795	9.58	21	2415.4	<0.001
(B) Sex ^b^	0.953	5.93	7	841.0	<0.001
(C) Generation ^c^	0.854	9.87	14	1682.0	<0.001
A × B	0.982	0.74	21	2415.4	0.794
A × C	0.886	2.45	42	3948.1	<0.001
B × C	0.970	1.84	14	1682.0	0.028
A × B × C	0.967	0.66	42	3948.1	0.953

Note: ^a^ a_1_, authoritative, a_2_, indulgent, a_3_, authoritarian, a_4_, neglectful; ^b^ b_1_, females, b_2_, males; ^c^ c_1_, first-generation children, c_2_, second-generation children, c_3_, third-generation children.

**Table 4:** Means and (standard deviations) of parenting style, generation, and sex, and main univariate *F* values for psychosocial adjustment (self-concept and well-being).

	Parenting Style				
	Authoritative	Indulgent	Authoritarian	Neglectful	*F*(3, 3847)
Self-Concept					
Academic/	8.27 ^a^	8.45 ^1^	7.83 ^2,b^	8.07 ^2^	10.09 ***
professional	(1.35)	(1.27)	(1.63)	(1.36)	
Social	7.31 ^1^	7.38 ^1^	6.39 ^2^	6.63 ^2^	22.30 ***
	(1.59)	(1.48)	(1.78)	(1.66)	
Emotional	5.73 ^2^	6.02 ^1^	5.65 ^2^	5.81 ^2^	3.04
	(2.09)	(2.20)	(2.10)	(1.99)	
Family	7.82 ^2^	8.25 ^1^	6.63 ^4^	7.25 ^3^	57.66 ***
	(1.33)	(1.29)	(1.72)	(1.77)	
Physical	5.10 ^1^	5.09 ^1^	4.29 ^2^	4.82 ^1^	11.48 ***
	(1.82)	(1.64)	(1.86)	(1.66)	
Well-Being					
Life Satisfaction	5.18 ^1^	5.21 ^1^	4.77 ^2^	4.88 ^2^	9.72 ***
	(1.20)	(1.14)	(1.18)	(1.16)	
Happiness	7.55 ^1^	7.51 ^1^	6.94 ^2^	7.08	6.01 ***
	(1.81)	(1.86)	(2.13)	(1.98)	

Note: Bonferroni Test α = 0.05; ^1^ > ^2^, ^a^ > ^b^. *** *p* < 0.001.

**Table 5:** Means and (standard deviations) of generation and sex, and main univariate *F* values for psychosocial adjustment (self-concept and well-being).

	Sex			Generation			
	Female	Male	*F*(1, 847)	G1	G2	G3	***F* (2, 847)**
Self-Concept							
Academic/	8.01	8.37	2.84	8.25 ^1^	8.33 ^1^	7.63 ^2^	19.49 ***
professional	(1.48)	(1.31)		(1.37)	(1.20)	(1.78)	
Social	6.93	(6.92	0.04	6.86	6.87	7.16	1.66
	(1.68)	(1.70)		(1.71)	(1.55)	(1.87)	
Emotional	5.43	6.38	22.37 ***	5.96 ^1^	5.80	5.51 ^2^	3.77 *
	(2.11)	(1.95)		(2.14)	(2.07)	(2.04)	
Family	7.58	7.35	5.51 *	7.48	7.41	7.64	0.99
	(1.69)	(1.59)		(1.51)	(1.50)	(2.13)	
Physical	4.77	4.91	2.41	4.41 ^2^	4.95 ^1^	5.31 ^1^	15.95 ***
	(1.73	(1.84)		(1.72)	(1.64)	(1.97)	
Well-Being							
Life Satisfaction	5.01	5.01	0.03	5.23 ^1^	4.99	4.92 ^2^	3.81 *
	(1.19)	(1.17)		(1.09)	(1.17)	(1.23)	
Happiness	7.36	7.14	0.29	7.98 ^1^	7.08 ^2^	7.08 ^2^	15.25 ***
	(1.91)	(2.04)		(1.40)	(2.03)	(2.08)	

Note: Bonferroni Test α = 0.05; ^1^ > ^2^ * *p* < 0.05, *** *p* < 0.001.
